# Population- and Sex-Biased Gene Expression in the Excretion Organs of *Drosophila melanogaster*

**DOI:** 10.1534/g3.114.013417

**Published:** 2014-09-22

**Authors:** Ann Kathrin Huylmans, John Parsch

**Affiliations:** Department of Biology II, University of Munich (LMU), 82152 Planegg, Germany

**Keywords:** malpighian tubule, transcriptome, sexual dimorphism, adaptation, RNA-seq

## Abstract

Within species, levels of gene expression typically vary greatly between tissues, sexes, individuals, and populations. To investigate gene expression variation between sexes and populations in a single somatic tissue, we performed a quantitative analysis of the Malpighian tubule transcriptome in adult males and females of *Drosophila melanogaster* derived from two distinct populations (one from sub-Saharan Africa and one from northern Europe). We identified 2308 genes that differed in expression between the sexes and 2474 genes that differed in expression between populations at a false discovery rate of 5%. We also identified more than 1000 genes that showed a sex-by-population interaction in their expression. The genes that differed in expression between sexes showed enrichment for a wide variety of functions, although only 55% of them overlapped with sex-biased genes identified in whole-fly studies. The genes expressed differentially between populations included several that were previously implicated in adaptive regulatory evolution, an excess of cytochrome P450 genes, and many genes that were not detected in previous studies of whole flies. Our results demonstrate that there is abundant intraspecific gene expression variation within in a single, somatic tissue and uncover new candidates for adaptive regulatory evolution between populations.

During the past 15 years, large-scale analyses of gene expression using microarrays or high-throughput RNA sequencing (RNA-seq) have revealed that there are abundant gene expression differences between tissues, developmental stages, sexes, individuals, and populations (Cavalieri *et al.* 2000; Jin *et al.* 2001; Arbeitman *et al.* 2002; Meiklejohn *et al.* 2003; Parisi *et al.* 2003; Ranz *et al.* 2003; Townsend *et al.* 2003; Chintapalli *et al.* 2007; Hutter *et al.* 2008; Brawand *et al.* 2011; Müller *et al.* 2011). In the model species *Drosophila melanogaster*, most previous studies of sex- or population-biased gene expression have focused on whole flies, body segments, or reproductive organs (Jin *et al.* 2001; Parisi *et al.* 2003; Ranz *et al.* 2003; Hutter *et al.* 2008; Müller *et al.* 2011; Meisel *et al.* 2012). Such studies provide a general overview of genes with broad expression or high expression in particular tissues. However, they lack the power to detect variation in weakly expressed, tissue-specific genes or genes that show opposing changes in expression between tissues. For these reasons, the analysis of specific tissues may uncover differences in gene expression that are overlooked in other studies (Catalán *et al.* 2012; Chintapalli *et al.* 2012; Johnson *et al.* 2013).

In the current study, we use RNA-seq to investigate gene expression variation in Malpighian tubules, which have a function analogous to that of human kidneys. The Malpighian tubules play a key role in osmoregulation and the excretion of waste products (Dow and Davies 2001; Wang *et al.* 2004). They are also important for the detoxification of xenobiotics, including insecticides (Torrie *et al.* 2004; Yang *et al.* 2007; Chahine and O’Donnell 2011). From an evolutionary and population genetics perspective, the Malpighian tubules of *D*. *melanogaster* are of interest because they are the major tissue of expression of several genes that show evidence for adaptive expression divergence between populations from the ancestral species range (sub-Saharan Africa) and those from derived, non-African habitats. For example, the cytochrome P450 gene *Cyp6g1*, which shows a large expression difference between African and European flies and confers insecticide resistance when overexpressed (Daborn *et al.* 2002; Hutter *et al.* 2008; Müller *et al.* 2011), displays its greatest expression in Malpighian tubules (Chintapalli *et al.* 2007). Similarly, the choline kinase gene *CG10560*, which is part of the four-gene *CHKov1* cluster that differs in expression between European and African flies and also has been implicated in insecticide resistance (Aminetzach *et al.* 2005; Catalán *et al.* 2012), has highly enriched expression in the Malpighian tubules (Chintapalli *et al.* 2007). Finally, the choline dehydrogenase gene *CG9505*, which shows evidence for an adaptive increase in expression in populations outside of sub-Saharan Africa (Saminadin-Peter *et al.* 2012; Glaser-Schmitt *et al.* 2013), has its greatest expression levels in the Malpighian tubules (Chintapalli *et al.* 2007).

Because *D*. *melanogaster* originated in sub-Saharan Africa and spread to other worldwide habitats only within the past 15,000 years (Li and Stephan 2006; Laurent *et al.* 2011; Duchen *et al.* 2013), the aforementioned findings suggest that gene regulatory changes in the Malpighian tubules might be particularly important during adaptation to new environments. To characterize population differentiation in gene expression, specifically in the Malpighian tubules, we sequenced the transcriptomes of flies derived from two populations, one from sub-Saharan Africa (Zimbabwe) and one from Europe (the Netherlands). Because gene expression often differs greatly between the sexes (Ellegren and Parsch 2007; Parsch and Ellegren 2013), we examined males and females separately. Overall, we find that there is a high amount of differential expression between sexes (2308 genes) and populations (2474 genes). The genes that differ in expression between populations include some of the candidates for adaptive regulatory evolution detected in previous studies using whole flies (*Cyp6g1*, *CG10560*, and *CG9509*), as well as many new genes that were not detected previously. Although most of the differentially expressed genes were consistent between sexes and populations, there were 615 genes that showed sex-biased expression in only one population and 557 genes that showed population-biased expression in only one sex. These findings indicate that there is abundant intraspecific gene expression variation within in a single, somatic tissue.

## Materials and Methods

### Fly strains and tissue preparation

Fly strains and rearing conditions were the same as those described in Catalán *et al.* (2012). In brief, we used 11 isofemale lines from sub-Saharan Africa (Lake Kariba, Zimbabwe) and 12 isofemale lines from Europe (Leiden, the Netherlands). All lines were maintained under common conditions (22°, cornmeal-molasses medium, light:dark cycle of 14 hr:10 hr) for more than 10 generations before RNA extraction.

**Figure 1:**
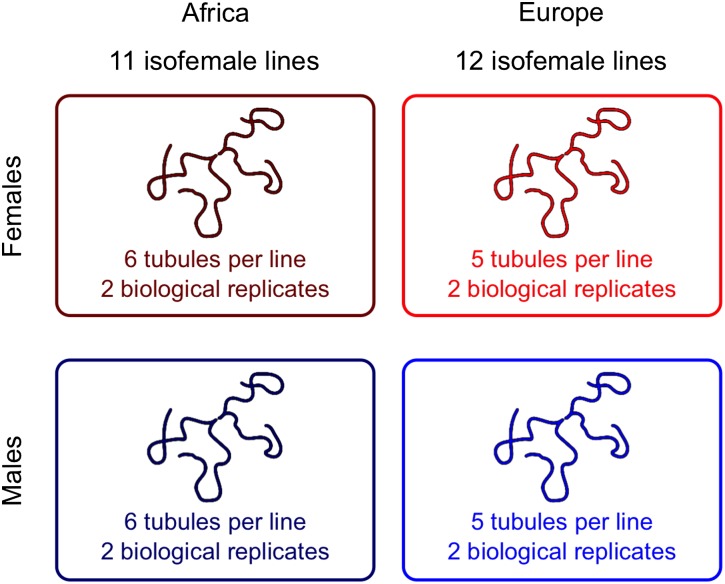
Overview of the experimental design. Malpighian tubules were dissected from multiple isofemale lines of each population and pooled for RNA extraction. Two biological replicates were performed for each sex and population, resulting in eight libraries that were used for sequencing.

Malpighian tubule dissections were performed on adult flies (4–6 d old) after anesthetizing them with CO_2_. At this age, expression profiles of Malpighian tubules have been shown to be stable in *D. melanogaster* (Wang *et al.* 2004). Dissections were done in 1× phosphate-buffered saline. The Malpighian tubules were cut at the lower ureter, and the part of the gut connecting the left and right tubule was not included. Dissected tissue was stored in RNAlater (QIAGEN, Hilden, Germany) and frozen at –80° until RNA extraction. To generate RNA pools representative of each population, six pairs of tubules from each of the 11 African lines (or five pairs from each of the 12 European lines) were combined for RNA extraction. The entire procedure was performed twice for each population and separately for males and females, resulting in a total of eight samples (two biological replicates of each population and sex). An overview of the experimental design is shown in Figure 1.

### RNA extraction and sequencing

Total RNA was extracted from dissected tissue using the MasterPure RNA Purification Kit (Epicentre, Madison, WI). RNA isolation was performed without DNase I digestion, because this was found to cause partial degradation of the RNA. Purification of mRNA, construction of cDNA libraries, and high-throughput sequencing were carried out by GATC Biotech (Konstanz, Germany). In brief, mRNA was enriched by poly(A) selection, sheared, and reverse-transcribed into cDNA using random hexamer primers. Eight individually tagged libraries were pooled and sequenced on one lane of a HiSequation 2500 (Illumina, San Diego, CA) to produce single-end reads of 50 bp. RNA extraction and library preparation were performed for all samples in parallel to avoid any day or batch effects. The sequence data have been deposited in NCBI’s Gene Expression Omnibus (Edgar *et al.* 2002) and are accessible through GEO series accession number GSE58578.

### Read mapping

Sequence reads were mapped to the *D. melanogaster* transcriptome (including noncoding RNAs) using the annotation of FlyBase release 5.54 (St. Pierre *et al.* 2014). Mapping of the raw reads was performed using NextGenMap (version 0.4.10) (Sedlazeck *et al.* 2013). To assess the influence of the mapping software on our results, we repeated the mapping with Stampy (version 1.0.22) (Lunter and Goodson 2011) and Bowtie2 (version 2.1.0) (Langmead and Salzberg 2012). Because the numbers of mapped reads per gene were highly correlated among mappers (Pearson’s *R* > 0.995 in all comparisons) and had only minor effects on the downstream analyses, only the results from NextGenMap are presented. Although the reference *D*. *melanogaster* genome is derived from a non-African lab strain, a previous simulation study using whole-genome sequences from African and European flies showed that this does not introduce a bias in the number of mapped reads per population (Catalán *et al.* 2012).

The expression analysis was performed in two ways: on a “per-gene” basis and on a “per-transcript” basis. For the former, NextGenMap was run with the default parameters and a read that mapped to any transcript isoform of a gene was counted as a “hit” to that gene. RPKM (reads per kilobase per million mapped reads) was calculated using the longest transcript of each gene. For the “per transcript” mapping, NextGenMap was run with its most sensitive settings (–kmer-skip 0 -s 0.0) to ensure that only reads with their best match to a single annotated transcript were included. Reads that had equal-quality matches to multiple transcripts were excluded. Because we had only 50-bp single-end reads, this approach greatly reduced the number of uniquely mapped reads and, thus, reduced statistical power to detect differential expression at the transcript level. For this reason, unless otherwise specified, all results refer to the “per-gene” analysis.

### Statistical analysis

Differentially expressed genes were detected using the Bioconductor (Gentleman *et al.* 2004) package DESeq2 (version 1.2.10) (Anders and Huber 2010) as implemented in R (version 3.0) (R Core Team 2014). A two-factor design with the factors population (Europe *vs.*. Africa) and sex (male *vs.* female) was used to analyze the eight samples. In this design, the fit of the data to a one-factor model is compared to that to a two-factor model to estimate the effect of each factor (sex and population) on gene expression. To adjust *P*-values for multiple testing and determine the false discover rate (FDR), Benjamini-Hochberg correction for multiple testing was used (Benjamini and Hochberg 1995). For the statistical analyses, we included only genes with a sufficient number of mapped reads for it to be possible to show a significant difference between sexes or populations after multiple test correction (Anders and Huber 2010). Given our study design and total read count, a minimum of 12 mapped reads was needed to reach an adjusted *P*-value below 5%. For example, a gene with three mapped reads in each of the four male libraries and zero mapped reads in each of the four female libraries (*i.e.*, 12 total reads) would give an uncorrected *P*-value of 0.002 and a multiple-test corrected *P*-value of 0.016. The numbers of analyzed and significant genes for other read-count thresholds are presented in Supporting Information, Table S1. To detect genes that differed in their sex bias between populations (or their population bias between sexes), the aforedescribed analysis was repeated using only data from a single population (or sex) and a one-factor model.

To assess how DESeq2 performs in comparison to other statistical methods, we also analyzed our data with edgeR (version 3.6.8) (McCarthy *et al.* 2012) and baySeq (version 1.18.0) (Hardcastle and Kelly 2010). Similar to the two-factor design in DESeq2, an interaction model for the factors population and sex with subsequent blocking of one of the factors was performed in edgeR. In baySeq, two differential expression models, one for population, the other for sex, were taken into account when estimating the posterior probabilities for all genes to be differentially expressed. For both methods, *P*-values were adjusted for multiple testing using the Benjamini-Hochberg correction (Benjamini and Hochberg 1995) and an FDR cut-off of 5% was applied. Both edgeR and baySeq identified fewer differentially expressed genes than DESeq2, although there was a high overlap between the significant genes identified by these methods and those identified by DESeq2 (Table S2). For this reason, we used the DESeq2 results for further downstream analyses.

### Functional enrichment analysis

Differentially expressed genes were functionally annotated using Gene Ontology (GO) terms (Ashburner *et al.* 2000). Statistical analysis of overrepresentation of terms within the differentially expressed genes in comparison to the whole genome was done with GOEAST (Zheng and Wang 2008) and confirmed with FlyMine (Lyne *et al.* 2007). FlyMine was also used to test for enrichment of protein domains. For both GOEAST and FlyMine, the Benjamini-Hochberg correction for multiple testing was applied (Benjamini and Hochberg 1995) and terms were considered significantly enriched if the adjusted *P*-value was below 5%. To account for hierarchical relationships among GO terms, only the most specific terms that were still significant are presented.

### Analysis of expression breadth

The expression breadth of population- and sex-biased genes was assessed by calculating *τ* (Yanai *et al.* 2005; Larracuente *et al.* 2008). Following Meisel (2011), we calculated *τ* for 14 adult tissues from FlyAtlas (Chintapalli *et al.* 2007). The tissues “head” and “carcass” were not included because they represent composite structures. The expression intensities for “spermatheca mated” and “spermatheca virgin” were averaged because they were shown to correlate well (Meisel 2011). If there were multiple microarray probes corresponding to the same gene, the probe with the greatest intensity in all tissues combined was used. Values of *τ* range from zero (housekeeping gene) to one (highly tissue-specific gene). In our analyses, genes with *τ* > 0.7 are considered narrowly expressed (Meisel *et al.* 2012; Gao *et al.* 2014).

## Results

We generated RNA-seq reads from Malpighian tubules dissected from adult males and females from an African and a European population of *D*. *melanogaster* (Figure 1). In total, there were 205 million reads, 89.4% of which could be mapped to the transcriptome. Most of the remaining reads mapped to ribosomal RNA (7.1%) or other noncoding RNA (0.5%), whereas 3.0% remained unmapped. Of the 13,942 annotated genes in FlyBase release 5.54 (St. Pierre *et al.* 2014), 12,547 had at least 12 mapped reads and were included in our statistical analysis (see the section *Materials and Methods*). The numbers of genes meeting greater read-count thresholds are presented in Table S1. There were 8231 genes that had an RPKM greater than one when averaged over all libraries. The RPKM values per gene were highly correlated between biological replicates (Pearson’s *R* > 0.98 in all cases).

### Sex-biased gene expression

**Figure 2:**
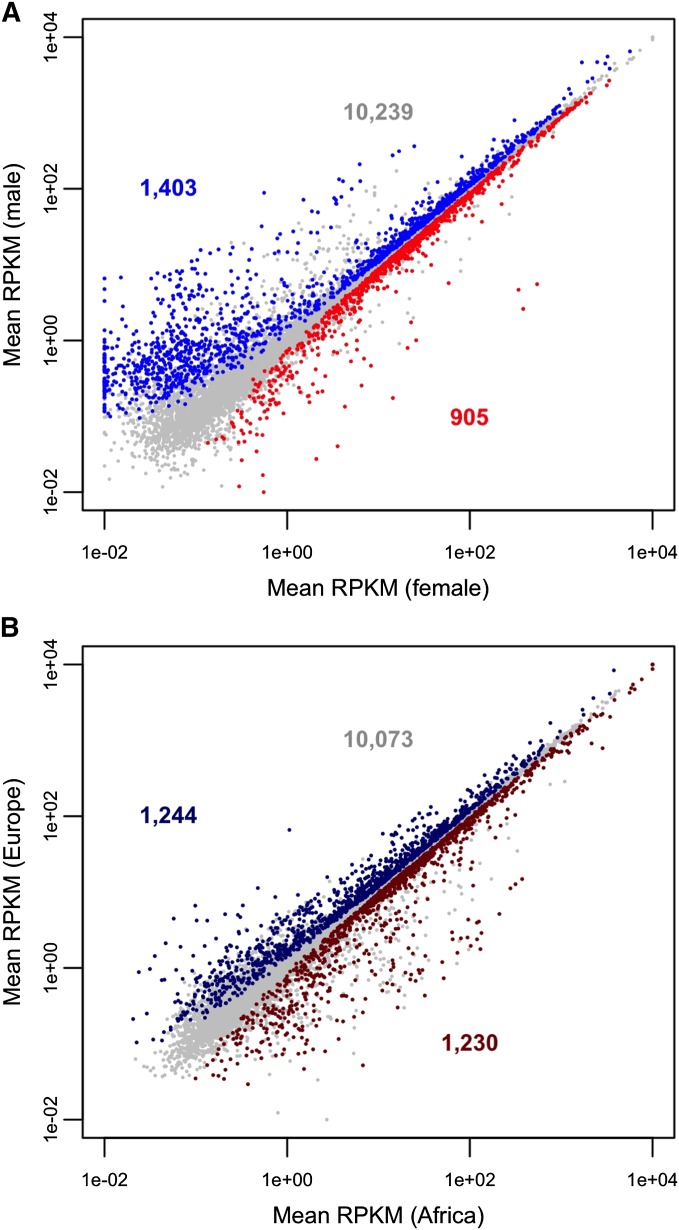
Differential gene expression between sexes and populations. (A) Comparison of gene expression between females and males. Blue points indicate male-biased genes, red points indicate female-biased genes, and gray points indicate genes with no sex bias. Expression values are from the combined analysis of both populations. The number of genes in each category is shown in the corresponding color. (B) Comparison of gene expression between the African and European populations. Dark blue points indicate Europe-biased genes, dark red points indicate Africa-biased genes, and gray points indicate genes with no population bias. Expression values are from the combined analysis of both sexes. The number of genes in each category is shown in the corresponding color. RPKM, reads per kilobase per million mapped reads.

In a two-factor (sex and population) analysis of the full data set, 2308 genes were detected as being differentially expressed between females and males at a false discovery rate (FDR) of 5% (Figure 2A; File S1). There were significantly more male-biased (1403) than female-biased (905) genes (sign test, *P* < 0.001). The male-biased genes also showed a greater degree of sex-bias: the median expression difference between the sexes was 2.13-fold for male-biased genes and 1.25-fold for female biased genes (Wilcoxon test, *P* < 0.001). However, male-biased genes tended to have lower overall expression than female-biased genes, with average RPKM values of 83 and 125, respectively. There was not a strong agreement between a gene’s sex-biased expression in the Malpighian tubules and in the whole fly (Gnad and Parsch 2006): 55% of the genes with sex-biased expression in Malpighian tubule showed the same sex bias in whole flies. Of the remaining genes with sex-biased expression in Malpighian tubule, 35% showed no sex bias in whole flies, whereas 10% showed the opposite sex bias.

Increasing the expression threshold for our statistical analysis led to a reduction in the number of sex-biased genes (Table S1). For example, when the minimum read-count threshold is increased from 12 to 100, the numbers of female- and male-biased genes are reduced by 12% and 24%, respectively. This indicates that many of the sex-biased genes, especially the male-biased genes, have an overall low expression level. However, even at this higher threshold, there were still significantly more male-biased (1073) than female-biased (798) genes (sign test, *P* < 0.001).

An analysis of GO terms revealed that female-biased genes were enriched with those involved in transport, energy metabolism, transcription, sex-determination, and cell division, whereas male-biased genes were enriched with those involved in lipid metabolism, glycolysis, and oxidation (Table 1, Table S3, and Table S4). The genes showing the strongest female-bias in their expression include three yolk protein genes, as well as several genes involved in pheromone metabolism and development (Table 2). The genes with the strongest male-biased expression included two genes known to be involved in male reproduction, although the majority of the highly male-biased genes were of unknown function (Table 2).

**Table 1:** Overrepresented biological process terms among sex-biased genes

Bias	GO Term	Genes	Adj. *P**^a^*
Female	Mitotic spindle elongation	24	5.83e-09
Female	ATP hydrolysis coupled proton transport	15	4.69e-07
Female	rRNA processing	14	1.29e-05
Female	Centrosome duplication	20	1.31e-05
Female	Germ cell development	44	0.0002
Female	Asymmetric neuroblast division	11	0.0016
Female	Regulation of cell shape	19	0.0042
Female	Regulation of growth	28	0.0048
Female	Actin cytoskeleton organization	27	0.0071
Female	Translational elongation	8	0.0084
Female	Chorion-containing eggshell formation	20	0.0114
Female	Long-term memory	12	0.0119
Female	ATP synthesis coupled proton transport	7	0.0119
Female	Ommochrome biosynthetic process	7	0.0119
Female	Regulation of cell differentiation	35	0.0134
Female	Mitotic cell-cycle phase transition	7	0.0212
Female	Pupariation	5	0.0267
Female	Regulation of DNA replication	6	0.0310
Male	Fatty acid beta-oxidation	10	4.63e-06
Male	Glutathione metabolic process	18	5.75e-06
Male	Protein folding	27	3.75e-05
Male	Glycolysis	12	0.0002
Male	Dicarboxylic acid metabolic process	10	0.0183
Male	Branched-chain amino acid metabolic process	5	0.0262
Male	Glycerol ether metabolic process	7	0.0326

a Multiple-test corrected *P*-value for term enrichment.

**Table 2:** Top 10 female- and male-biased genes

Gene	Bias	Log_2_(M/F)*^a^*	Adj. *P**^b^*	Biological Process
*dhd*	Female	**-**5.85	3.0e-44	Cellular response to DNA damage stimulus
*Yp3*	Female	−5.63	1.9e-47	Embryo development
*Btd*	Female	−5.08	3.2e-33	Response to heat
*Fad2*	Female	−4.56	1.8e-20	Pheromone metabolic process
*Obp99a*	Female	−4.12	2.3e-27	Response to pheromone
*Yp2*	Female	−4.11	3.5e-31	Neurogenesis
*fit*	Female	−3.25	2.9e-53	Unknown
*eloF*	Female	−3.11	5.0e-13	Pheromone metabolic process
*osk*	Female	−2.87	3.5e-14	Germ cell development
*Yp1*	Female	−2.55	1.1e-06	Vitellogenesis
*CG3124*	Male	5.40	1.6e-32	Unknown
*Mst84Db*	Male	5.24	3.4e-29	Spermatogenesis
*CG8701*	Male	5.24	3.4e-31	Unknown
*CG12861*	Male	4.79	9.3e-26	Unknown
*CG12860*	Male	4.69	4.8e-30	Unknown
*S-Lap1*	Male	4.67	1.8e-23	Proteolysis
*Met75Ca*	Male	4.64	3.4e-21	Multicellular organism reproduction
*CG9130*	Male	4.60	1.2e-20	Unknown
*CG4836*	Male	4.55	1.8e-20	Oxidation-reduction process
*CG9016*	Male	4.54	3.7e-21	Unknown

a Log_2_(male expression/female expression).

b Multiple-test corrected *P*-value for differential expression between the sexes.

In addition to the gene-based analyses, we also investigated sex-biased expression at the level of individual transcripts. For this, we included only RNA-seq reads that could be mapped unambiguously to a specific transcript of a gene with multiple annotated transcripts. In total, we were able to map 66.5 million reads to 13,482 transcripts of 6594 multiple-transcript genes. Although the lower number of mapped reads reduced our statistical power to detect differential expression in comparison to the gene-based analysis, we were still able to detect 230 transcripts from 207 genes that differed in expression between females and males at a FDR of 5% (File S2). The vast majority of these were cases in which only a single transcript of a gene showed significant differential expression between the sexes (186 transcripts) or multiple transcripts of a gene showed a significant bias toward the same sex (38 transcripts). The former group included genes known to be involved in sex determination, such as *Sex lethal* and *doublesex*, as well as seven ribosomal protein genes (*RpL17*, *RpL37a*, *RpLS14a*, *RpS15Aa*, *RpS19a*, *RpS2*, and *RpS28b*). There were also three genes (*RpL35*, *sesB*, and *regucalcin*) that had one transcript with significantly female-biased expression and another transcript with significantly male-biased expression.

### Population-biased gene expression

A total of 2474 genes showed consistent, significant differences in their level of Malpighian tubule expression between the African and European populations in both sexes (Figure 2B; File S1). There was no significant difference between the number of Africa-biased (1230) and Europe-biased (1244) genes (sign test, *P* = 0.79). However, the degree of population bias was slightly higher for Europe-biased (1.42-fold) than for Africa-biased (1.28-fold) genes (Wilcoxon test, *P* < 0.001). Increasing the expression threshold for our statistical analysis led to a slight reduction in the number of population-biased genes (Table S1). For example, when the minimum read-count threshold is increased from 12 to 100, the numbers of Africa- and Europe-biased genes are reduced by 3.9% and 3.5%, respectively. This indicates that the vast majority of the population-biased genes have a relatively high overall level of expression.

Among the genes that differed in expression between the African and European populations, there was a significant excess of those encoding proteins with conserved cytochrome P450 domains (40 genes, *P* = 0.0002). Of these genes, there were similar numbers showing overexpression in Africa (22 genes) and Europe (18 genes). The GO categories overrepresented among the genes with significantly greater expression in Africa indicate that these genes, in addition to playing roles in transport and energy metabolism that are expected for the Malpighian tubules, are also involved in male courtship behavior, morphogenesis, and growth (Table 3; Table S5). Genes with functions in stress response and centrosome organization were more highly expressed in the Malpighian tubules of European flies than of African flies (Table 3; Table S6). The genes with the greatest overexpression in African flies included those with annotated functions in the nervous system and perception, whereas the genes with the greatest overexpression in European flies included mainly those involved in metabolism (Table 4).

**Table 3:** Overrepresented biological process terms among population-biased genes

Bias	GO Term	Genes	Adj. *P**^a^*
Africa	ATP hydrolysis coupled proton transport	16	7.50e-06
Africa	Carbohydrate metabolic process	48	0.0001
Africa	Male courtship behavior	10	0.0006
Africa	Chemical homeostasis	25	0.0008
Africa	Negative regulation of signal transduction	35	0.0008
Africa	Neuron development	83	0.0012
Africa	Regulation of organ morphogenesis	19	0.0041
Africa	Glutathione metabolic process	13	0.0074
Africa	Visual perception	11	0.0158
Africa	Taxis	43	0.0174
Africa	Regulation of transport	24	0.0197
Africa	Dorsal/ventral pattern formation	28	0.0207
Africa	Regulation of immune system process	23	0.0240
Europe	Oxidation-reduction process	87	4.57e-08
Europe	Organonitrogen compound metabolic process	84	0.0006
Europe	Carboxylic acid metabolic process	49	0.0007
Europe	Carbohydrate derivative metabolic process	59	0.0075
Europe	Cellular response to stress	56	0.0233
Europe	Centrosome organization	23	0.0343

a Multiple-test corrected *P*-value for term enrichment.

**Table 4:** Top 10 Africa- and Europe-biased genes

Gene	Bias	Log_2_(E/A)^a^	Adj. *P*^b^	Biological Process
*CG6475*	Africa	−4.71	4.0e-37	Metabolic process
*CG13889*	Africa	−4.50	5.2e-115	Olfactory behavior
*mtg*	Africa	−3.89	4.3e-29	Synapse assembly
*CG10508*	Africa	−3.84	7.3e-210	Unknown
*CG12438*	Africa	−3.19	1.3e-17	Unknown
*CG10257*	Africa	−2.85	7.4e-13	Neuron projection morphogenesis
*CG15414*	Africa	−2.78	2.5e-13	Unknown
*Gr61a*	Africa	−2.73	2.5e-13	Sensory perception of sweet taste
*CG14110*	Africa	−2.64	5.3e-18	Unknown
*Gr22a*	Africa	−2.60	4.5e-12	Sensory perception of taste
*CG11697*	Europe	5.58	1.5e-205	Unknown
*CG13654*	Europe	4.42	3.4e-36	Unknown
*CG32506*	Europe	4.05	9.6e-41	Regulation of Rab GTPase activity
*CG31157*	Europe	3.96	2.7e-50	Unknown
*CG10924*	Europe	3.45	1.5e-19	Gluconeogenesis
*CG12934*	Europe	3.43	5.3e-23	Unknown
*ppk5*	Europe	3.40	3.3e-18	Sodium ion transport
*CG12951*	Europe	3.39	6.8e-40	Proteolysis
*CG4927*	Europe	3.38	6.7e-162	Proteolysis
*CG11313*	Europe	3.36	1.1e-16	Hemolymph coagulation

a Log_2_(Europe expression/Africa expression).

b Multiple-test corrected *P*-value for differential expression between the populations.

At the transcript level, we detected 511 transcripts from 468 genes that differed in expression between the African and European populations at a FDR of 5% (File S2). The vast majority of these were cases in which only a single transcript of a gene showed significant differential expression between the populations (430 transcripts) or multiple transcripts of a gene showed a significant bias toward the same population (65 transcripts). There were also seven genes (*CG1637*, *CG5697*, *CG10320*, *CG13565*, *be*, *DAAM*, and *Wbp2*), mostly of unknown function, that had one transcript with significantly Africa-biased expression and another transcript with significantly Europe-biased expression.

### Overlap of sex- and population-biased genes

**Figure 3:**
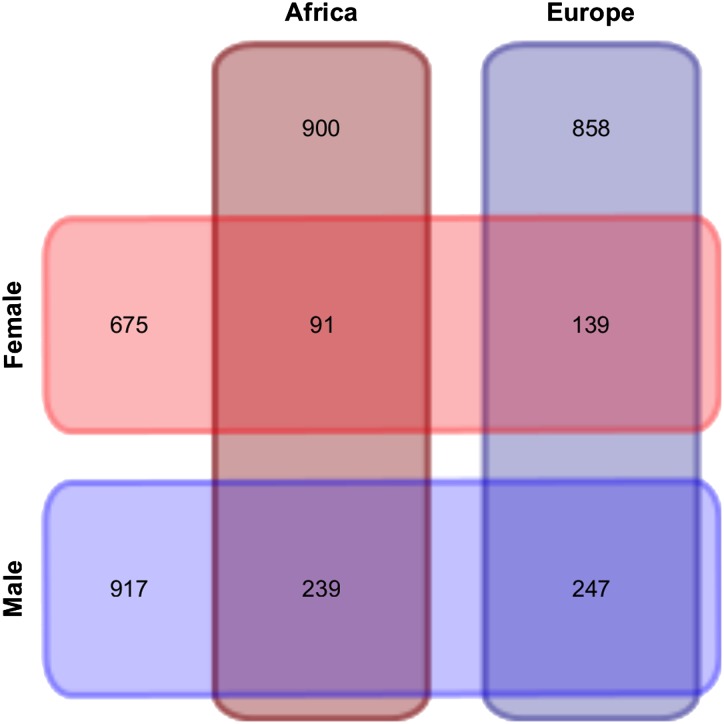
Venn diagram showing numbers of differentially expressed genes between sexes and populations, as well as their overlap.

Of the 2308 genes that showed sex-biased expression in our two-factor analysis, 716 (31%) also showed population-biased expression (Figure 3). A greater proportion of male-biased genes (35%) than female-biased (25%) genes showed differential expression between the populations (Fisher exact test, *P* < 0.001). Interestingly, nine of the 10 genes with the greatest male bias also differed in expression between populations, whereas none of the 10 genes with the greatest female bias differed in expression between populations (Fisher exact test, *P* = 0.0001). This pattern held for larger sets of genes: 45 of the top 100 male-biased genes showed population-biased expression, whereas only 10 of the top 100 female-biased genes showed population-biased expression (Fisher exact test, *P* < 0.0001). These observations indicate that male-biased genes, especially those with strong male bias, are more likely to differ in expression between populations than female-biased genes. Of the 2474 genes that showed population-biased expression in our two-factor analysis, 716 (29%) also showed sex-biased expression (Figure 3). A slightly greater proportion of Europe-biased genes (31%) than Africa-biased (27%) genes showed differential expression between the sexes (Fisher exact test, *P* = 0.024).

### Sex- and population-specific differential expression

**Figure 4:**
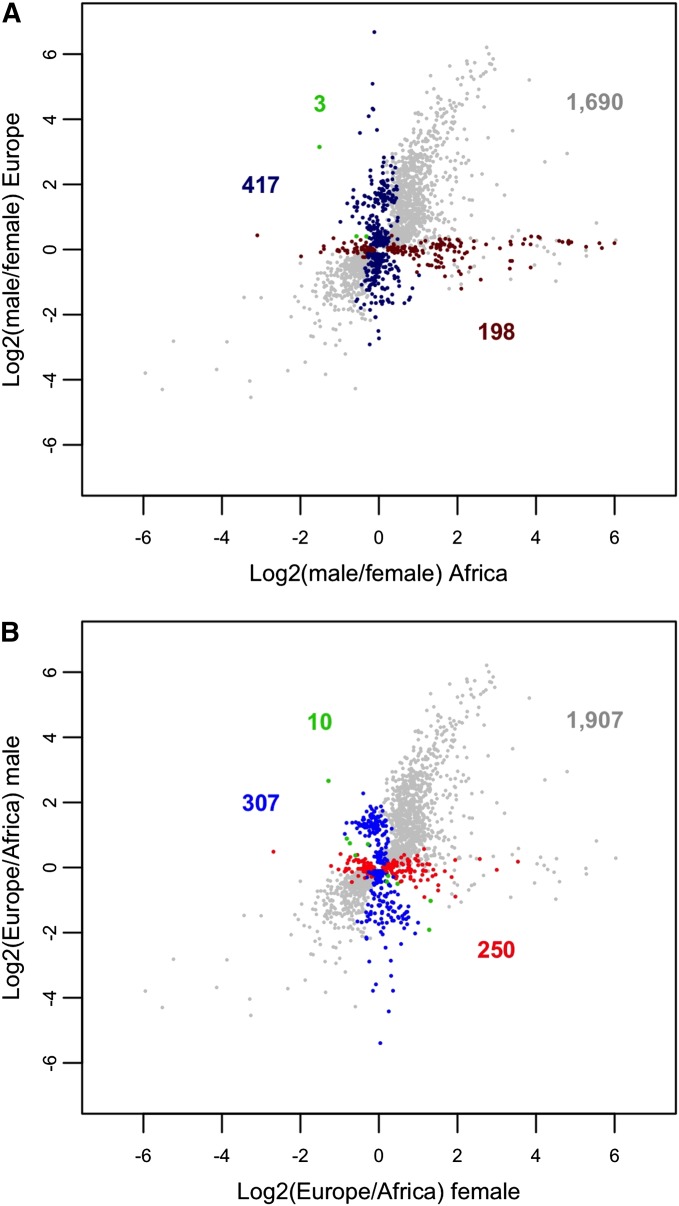
Expression differences by sex and population. (A) Gray points indicate genes with significantly sex-biased expression in the combined analysis of both populations. Dark blue points indicate genes that were sex-biased only in the European population, whereas dark red points indicate genes that were sex-biased only in the African population. Green points indicate genes with conflicting sex bias between the two populations. The number of genes in each category is shown in the corresponding color. (B) Gray points indicate genes with significantly population-biased expression in the combined analysis of both sexes. Blue points indicate genes that were population-biased only in males, while red points indicate genes that were population-biased only in females. Green points indicate genes with conflicting population bias between the two sexes. The number of genes in each category is shown in the corresponding color.

In addition to the 2308 genes detected as sex-biased in the joint analysis of the African and European populations, we found 198 genes with sex-biased expression only in the African population (68 female-biased and 130 male-biased; Figure 4A). Among these genes, the only significant GO term enrichment was for the female-biased genes, which showed enrichment for the biological process “antimicrobial humoral response” (15 genes, *P* = 0.0019) and the molecular function “structural constituent of ribosome” (17 genes, *P* = 0.0015). There were also 417 genes with sex-biased expression only in the European population (196 female-biased and 221 male-biased; Figure 4A). Of these, the male-biased genes showed enrichment for the biological process “cellular response to heat” (six genes, *P* = 8.33e^-4^) and the molecular function “endopeptidase inhibitor activity” (nine genes, *P* = 0.0025). We also found three genes of unknown function (*CG7225*, *CG31643*, and *CG17018*) that showed conflicting sex bias between the populations (all were male-biased in Africa, but female-biased in Europe).

There were 2474 genes that differed in expression between the populations in the combined analysis of both sexes. In addition to these, we found 250 genes with population-biased expression that was limited to females (138 Africa-biased and 112 Europe-biased; Figure 4B). Among these genes, those with an African bias showed enrichment for the biological processes “response to stimulus” (53 genes, *P* = 0.0061) and “response to chemical” (26 genes, *P* = 0.0065). We also found 307 genes with population-biased expression that was limited to males (153 Africa-biased and 154 Europe-biased; Figure 4B). These genes did not show any enrichment of GO terms. There were an additional 10 genes that differed in their population bias between females and males. Of these, five were Africa-biased in females, but Europe-biased in males (*Btd*, *Cda5*, *CG14291*, *CG14868*, *wbl*) and five were Europe-biased in females, but Africa-biased in males (*5-HT2B*, *aay*, *CG9279*, *CG13800*, *fat-spondin*).

### Expression breadth of differentially expressed genes

To investigate the tissue specificity of genes expressed differently between sexes or populations, we calculated the statistic *τ*, which ranges from zero (housekeeping gene) to one (highly tissue-specific gene) (Yanai *et al.* 2005; Larracuente *et al.* 2008). This revealed that male-biased genes had greater tissue-specificity than genes without sex-biased expression, while female-biased genes had less (Wilcoxon test, *P* < 0.0001 in both cases) (Table 5). However, the increased tissue specificity of male-biased genes was not attributable to genes that have their highest expression in Malpighian tubule, but instead to genes that have their highest expression in testis (Table 5). In terms of Malpighian tubule-specific expression, both the sex- and population-biased genes showed a significant excess of narrowly expressed genes with their highest expression in Malpighian tubule relative to non-biased genes (Wilcoxon test, *P* < 0.0001 in all cases). However, such genes represented only a small minority (1–2%) of all genes with sex- or population-biased expression (Table 5).

**Table 5:** Expression breadth of differentially expressed genes

Genes	Number	Median *τ*	Narrow*^a^* (%)	Tubule*^b^* (%)	Testis*^c^* (%)
All	13,689	0.38	2346 (16%)	71 (0.5%)	1219 (8.9%)
Female-biased	905	0.26	72 (8%)	10 (1.1%)	5 (0.6%)
Male-biased	1403	0.61	525 (37%)	16 (1.1%)	446 (32%)
Africa-biased	1230	0.32	141 (11%)	18 (1.5%)	15 (1.2%)
Europe-biased	1244	0.35	164 (13%)	19 (1.5%)	97 (7.8%)

a Genes with narrow expression breadth (*τ* > 0.7).

b Genes with narrow expression breadth and greatest expression in Malpighian tubule.

c Genes with narrow expression breadth and greatest expression in testis.

## Discussion

Although the Malpighian tubule is a somatic tissue shared by females and males, it displays a considerable number of genes that are expressed differentially between the sexes. Of the genes with sufficient expression for statistical analysis, 18.4% differed in expression between the sexes. In contrast, a previous study using the same experimental design found much less sex-biased expression in the brain, where only 0.8% of the genes differed in expression between the sexes (Catalán *et al.* 2012). To better compare the two studies, we re-analyzed the Catalán *et al.* (2012) RNA-seq data using the same genome annotation, mapping software, and statistical methods that we applied to the Malpighian tubule data (see *Materials and Methods*). This led to an increase in the proportion of genes detected as sex-biased in the brain (2.4%), but it was still much lower than in the Malpighian tubules. There were also more genes with highly sex-biased expression in the Malpighian tubules: 848 genes showed greater than a twofold expression difference between the sexes in the Malpighian tubules (130 female-biased and 718 male-biased), whereas only 50 genes showed greater than a twofold expression difference between the sexes in the brain (23 female-biased and 27 male-biased). Thus, despite the conspicuous behavioral differences between females and males (*e.g.*, courtship behavior), there appears to be much less sexually dimorphic gene expression in the brain than in other tissues.

In terms of sex-biased expression, our results are generally concordant with those of a previous microarray study that examined Malpighian tubule expression in males and females of a single laboratory strain (Chintapalli *et al.* 2012), with 42% of the female-biased genes and 58% of the male-biased genes identified in their study showing the same sex bias in ours. Thus, there is compelling evidence that sexual dimorphism in gene expression is maintained in this somatic tissue. Differences between the two studies may be related to differences in the methodologies (microarrays *vs.* RNA-seq) or experimental designs (a single laboratory strain *vs.* pooled strains from two natural populations) that were employed. Consistent with the latter, we found 615 genes for which sex-biased expression was observed in only one of our two populations. This finding suggests that the differences between the studies may reflect natural intraspecific variation in gene expression. Further differences may be attributable to the increased sensitivity that oligonucleotide-based microarrays have to detect isoform-specific expression of transcripts, which was limited in our RNA-seq analysis.

Chintapalli *et al.* (2012) found that the receptors for sex peptide and neuropeptide F showed male-biased expression in the tubule and hypothesized that they might be involved in neuropeptide control and stress response. The latter is supported by a study by Wen *et al.* (2005), who found that interruption of this signaling pathway is linked to increased ethanol tolerance. In our study, we also observed male-biased expression of the sex peptide receptor and neuropeptide F receptor 1, but not of the short neuropeptide F receptor. However, both sex peptide receptor and neuropeptide F receptor 1 also show significantly greater expression in Europe than in Africa, which indicates that the differential expression of the neuropeptide F receptor in the Malpighian tubules (fivefold greater in Europe) might contribute to the observed difference in ethanol tolerance between tropical and temperate flies (David *et al.* 1986).

Overall, we found that 19.7% of genes differed in their Malpighian tubule expression between a population from the ancestral species range in sub-Saharan Africa and one from the derived species range in Europe. This finding suggests that gene regulation has changed considerably in response to local environmental conditions. Furthermore, because we used a “common garden” experimental design in which flies from both populations were raised under identical conditions for multiple generations prior to RNA extraction, we can assume that the differences in expression have an underlying genetic basis and are not the result of phenotypic plasticity. Several of the genes that were previously identified as showing expression divergence between populations in whole-fly studies were also detected as differing in expression between populations in the Malpighian tubule. These include *Cyp6g1* (2.1-fold overexpression in Europe), *CG10560* (2.4-fold overexpression in Africa), and *CG9509* (1.9-fold overexpression in Europe), all of which show functional and population genetic evidence for recent adaptive evolution (Daborn *et al.* 2002; Catania *et al.* 2004; Aminetzach *et al.* 2005; Magwire *et al.* 2011; Catalán *et al.* 2012; Saminadin-Peter *et al.* 2012; Glaser-Schmitt *et al.* 2013). This suggests that some of the other population-biased genes also may have undergone adaptive regulatory evolution. Thus, our study has revealed new candidate genes for further functional and population genetic analyses. In this context, it is interesting to note the significant enrichment of cytochrome P450 genes among those that differ in expression between the populations (22 Africa-biased and 18 Europe-biased), which may result from selective pressure to detoxify different chemicals encountered in the two environments. The cytochrome P450 gene *Cyp6g1*, which confers insecticide resistance when overexpressed, is a well-documented example of such adaptation (Daborn *et al.* 2002; Catania *et al.* 2004).

It is also noteworthy that genes involved in the innate immune response are enriched among those showing population-specific differences in sex-biased expression, with there being an excess of antimicrobial genes with female-biased expression in the African population. The Malpighian tubules are known to play an important role in immune defense (Tzou *et al.* 2002; McGettigan *et al.* 2005) and it was previously shown that immune genes are expressed differently between the sexes in Malpighian tubules (Chintapalli *et al.* 2012), suggesting that females and males face distinct immune challenges. Our results indicate that these immune challenges also may differ between populations and that the innate immune response evolves largely independently in the two sexes.

## Supplementary Material

Supporting Information
