# Supplementary material for: Population- and Sex-Biased Gene Expression in the Excretion Organs of Drosophila melanogaster
Source: G3 (Bethesda). 2014 Sep 22;4(12):2307–15. doi: 10.1534/g3.114.013417 (PMC4267927; doi:10.1534/g3.114.013417)
Supplement: Supporting Information [file supp_4_12_2307__index.html]

Population- and Sex-Biased Gene Expression in the Excretion Organs of Drosophila melanogaster — Supporting Information 

# Population- and Sex-Biased Gene Expression in the Excretion Organs of *Drosophila melanogaster*

## Supporting Information for Huylmans and Parsch, 2014

**Files in this Data Supplement:**

- Supporting Information - Files S1-S2 and Tables S1-S6 (PDF, 179 KB)
- Table S1 - Number of genes meeting various read-count thresholds. (PDF, 100 KB)
- Table S2 - Number of significant genes detected by different methods. (PDF, 100 KB)
- Table S3 - Over-represented GO terms among female-biased genes. (PDF, 119 KB)
- Table S4 - Over-represented GO terms among male-biased genes. (PDF, 116 KB)
- Table S5 - Over-represented GO terms among Africa-biased genes. (PDF, 118 KB)
- Table S6 - Over-represented GO terms among Europe-biased genes. (PDF, 116 KB)
- File S1 - Tab-delimited text file containing gene ID, mapped read count, RPKM, *P*-value, and FDR for each gene compared between sexes and populations. Populations are abbreviated as "A" (Africa) or "E" (Europe). Sexes are abbreviated as "F" (female) or "M" (male). (.txt, 4 MB)
- File S2 - Excel spreadsheet containing transcript ID, gene ID, mapped read count, RPKM, *P*-value, and FDR for transcript isoforms showing a significant expression difference between sexes (worksheet 1) or populations (worksheet 2). Populations are abbreviated as "A" (Africa) or "E" (Europe). Sexes are abbreviated as "F" (female) or "M" (male). (.xls, 317 KB)
