# Supplementary material for: Population- and Sex-Biased Gene Expression in the Excretion Organs of Drosophila melanogaster
Source: G3 (Bethesda). 2014 Sep 22;4(12):2307–15. doi: 10.1534/g3.114.013417 (PMC4267927; doi:10.1534/g3.114.013417)
Supplement: Supporting Information [file supp_g3.114.013417_013417SI.pdf]

G3 · Genes | Genomes | Genetics

Population- and sex-biased gene expression in the excretion organs of *Drosophila melanogaster*

Ann Kathrin Huylmans and John Parsch*

Department of Biology II, University of Munich (LMU), 82152 Planegg, Germany

*Corresponding author: LMU Biocenter, Grosshaderner Str. 2, 82152 Planegg, Germany, E-mail: parsch@bio.lmu.de

DOI: 10.1534/g3.114.013417

File S1

Tab-delimited text file containing gene ID, mapped read count, RPKM, *P*-value, and FDR for each gene compared between sexes and populations. Populations are abbreviated as "A" (Africa) or "E" (Europe). Sexes are abbreviated as "F" (female) or "M" (male).

Available for download at http://www.g3journal.org/lookup/suppl/doi:10.1534/g3.114.013417/-/DC1

File S2

Excel spreadsheet containing transcript ID, gene ID, mapped read count, RPKM, *P*-value, and FDR for transcript isoforms showing a significant expression difference between sexes (worksheet 1) or populations (worksheet 2). Populations are abbreviated as "A" (Africa) or "E" (Europe). Sexes are abbreviated as "F" (female) or "M" (male).

Available for download at http://www.g3journal.org/lookup/suppl/doi:10.1534/g3.114.013417/-/DC1

Table S1 Number of genes meeting various read-count thresholds

Reads	Genes	Sex-biased (female, male)	Population-biased (Africa, Europe)
12	12,547	2,308 (905, 1,403)	2,474 (1,230, 1,244)
20	12,001	2,272 (898, 1,374)	2,459 (1,222, 1,237)
50	10,793	2,098 (858, 1,240)	2,436 (1,206, 1,230)
100	9,785	1,871 (798, 1,073)	2,382 (1,182, 1,200)
150	9,272	1,785 (772, 1,013)	2,340 (1,164, 1,176)

Shown is the total number of genes meeting each read-count threshold, as well as the number of significant sex- and population-biased genes at a FDR of 5%.

Table S2 Number of significant genes detected by different methods

Bias	DESeq2	edgeR (overlap[a])	baySeq (overlap[a])
Sex	2,308	1,249 (85.6%)	557 (98.4%)
Female	905	251 (91.2%)	88 (98.9%)
Male	1,403	998 (84.2%)	469 (98.3%)
Population	2,474	1,015 (87.9%)	496 (97.2%)
Africa	1,230	465 (88.2%)	200 (95.5%)
Europe	1,244	550 (87.6%)	296 (98.3%)

[a] Percent overlap with the significant genes detected by DESeq2.

Table S3 Over-represented GO terms among female-biased genes.

Ontology	GO Term	Genes	Adj. *P*
Molecular function	structural constituent of ribosome	54	1.8e-12
Molecular function	proton-transporting ATPase activity, rotational mechanism	15	1.1e-08
Molecular function	ATP binding	70	9.6e-05
Molecular function	mRNA binding	29	0.001
Molecular function	proton-transporting ATP synthase activity, rotational mechanism	7	0.001
Molecular function	structural constituent of chorion	6	0.001
Molecular function	ATP-dependent RNA helicase activity	11	0.003
Molecular function	hydrogen-exporting ATPase activity, phosphorylative mechanism	8	0.005
Molecular function	actin binding	19	0.01
Molecular function	microtubule binding	14	0.02
Molecular function	aminoacyl-tRNA editing activity	4	0.02
Molecular function	L-amino acid transmembrane transporter activity	5	0.03
Molecular function	symporter activity	14	0.03
Molecular function	protein serine/threonine kinase activity	25	0.03
Molecular function	inorganic anion transmembrane transporter activity	11	0.04
Molecular function	acid phosphatase activity	5	0.04
Cellular component	cytosolic large ribosomal subunit	22	6.0e-10
Cellular component	cytosolic small ribosomal subunit	19	6.0e-10
Cellular component	plasma membrane proton-transporting V-type ATPase complex	10	1.6e-07
Cellular component	microtubule associated complex	50	6.8e-06
Cellular component	lipid particle	34	1.8e-05
Cellular component	vacuolar proton-transporting V-type ATPase, V1 domain	7	0.001
Cellular component	chorion	11	0.001
Cellular component	spindle midzone	6	0.002
Cellular component	nucleolus	21	0.003
Cellular component	mitochondrial matrix	23	0.006
Cellular component	mitochondrial proton-transporting ATP synthase complex, catalytic core F(1)	5	0.01
Cellular component	small-subunit processome	4	0.02
Cellular component	euchromatin	6	0.03
Cellular component	centrosome	14	0.03
Biological process	mitotic spindle elongation	24	5.8e-09
Biological process	ATP hydrolysis coupled proton transport	15	4.7e-07
Biological process	rRNA processing	14	1.3e-05
Biological process	centrosome duplication	20	1.3e-05
Biological process	germ cell development	44	0.0002
Biological process	protein localization	49	0.002
Biological process	asymmetric neuroblast division	11	0.002
Biological process	regulation of cell shape	19	0.004

Biological process	regulation of growth	28	0.005
Biological process	ribosomal small subunit biogenesis	5	0.005
Biological process	protein phosphorylation	33	0.006
Biological process	negative regulation of cellular process	78	0.007
Biological process	actin cytoskeleton organization	27	0.007
Biological process	female germ-line sex determination	4	0.008
Biological process	translational elongation	8	0.008
Biological process	regulation of cellular component size	17	0.01
Biological process	chorion-containing eggshell formation	20	0.01
Biological process	cellular macromolecule localization	39	0.01
Biological process	long-term memory	12	0.01
Biological process	ATP synthesis coupled proton transport	7	0.01
Biological process	ommochrome biosynthetic process	7	0.01
Biological process	regulation of cell differentiation	35	0.01
Biological process	regulation of cellular component biogenesis	22	0.02
Biological process	regulation of cytoskeleton organization	15	0.02
Biological process	L-amino acid transport	4	0.02
Biological process	mitotic cell cycle phase transition	7	0.02
Biological process	establishment of localization in cell	53	0.02
Biological process	pupariation	5	0.03
Biological process	regulation of DNA replication	6	0.03
Biological process	cellular component assembly involved in morphogenesis	23	0.03
Biological process	amino acid transmembrane transport	8	0.03
Biological process	positive regulation of cellular component organization	16	0.03

Table S4 Over-represented GO terms among male-biased genes.

Ontology	GO Term	Genes	Adj. *P*
Molecular function	electron carrier activity	42	5.2e-10
Molecular function	manganese ion binding	12	4.1e-06
Molecular function	iron ion binding	37	5.4e-06
Molecular function	glutathione transferase activity	17	7.3e-06
Molecular function	acyl-CoA dehydrogenase activity	10	2.2e-05
Molecular function	oxidoreductase activity, acting on paired donors, with incorporation or reduction of molecular oxygen	37	4.6e-05
Molecular function	flavin adenine dinucleotide binding	19	5.4e-05
Molecular function	oxidoreductase activity, acting on the CH-OH group of donors, NAD or NADP as acceptor	23	7.5e-05
Molecular function	NAD binding	13	0.003
Molecular function	aminopeptidase activity	13	0.01
Molecular function	carbohydrate kinase activity	8	0.01
Molecular function	hydro-lyase activity	13	0.02
Molecular function	oxidoreductase activity, acting on the aldehyde or oxo group of donors, NAD or NADP as acceptor	11	0.02
Molecular function	long-chain fatty acid transporter activity	5	0.03
Molecular function	disulfide oxidoreductase activity	10	0.03
Cellular component	lipid particle	44	5.9e-06
Cellular component	mitochondrial matrix	35	2.6e-05
Cellular component	endoplasmic reticulum part	23	0.02
Cellular component	coated vesicle membrane	9	0.03
Cellular component	peroxisome	12	0.03
Cellular component	Golgi membrane	11	0.04
Biological process	fatty acid beta-oxidation	10	4.6e-06
Biological process	glutathione metabolic process	18	5.8e-06
Biological process	protein folding	27	3.8e-05
Biological process	glycolysis	12	0.0002
Biological process	dicarboxylic acid metabolic process	10	0.02
Biological process	branched-chain amino acid metabolic process	5	0.03
Biological process	glycerol ether metabolic process	7	0.03

Table S5 Over-represented GO terms among Africa-biased genes.

Ontology	GO Term	Genes	Adj. *P*
Molecular function	proton-transporting ATPase activity, rotational mechanism	15	1.7e-06
Molecular function	heme binding	30	7.3e-05
Molecular function	iron ion binding	30	0.002
Molecular function	anion binding	117	0.002
Molecular function	cofactor binding	30	0.01
Molecular function	nucleotide binding	122	0.02
Molecular function	protein kinase activity	40	0.04
Molecular function	oxidoreductase activity, acting on the CH-OH group of donors, NAD or NADP as acceptor	17	0.04
Molecular function	glutathione transferase activity	11	0.04
Cellular component	vacuolar proton-transporting V-type ATPase, V1 domain	9	8.9e-05
Biological process	ATP hydrolysis coupled proton transport	16	7.5e-06
Biological process	carbohydrate metabolic process	48	0.0001
Biological process	male courtship behavior, veined wing generated song production	10	0.001
Biological process	chemical homeostasis	25	0.001
Biological process	negative regulation of signal transduction	35	0.001
Biological process	neuron development	83	0.001
Biological process	regulation of organ morphogenesis	19	0.004
Biological process	glutathione metabolic process	13	0.007
Biological process	regulation of neurogenesis	24	0.01
Biological process	visual perception	11	0.02
Biological process	taxis	43	0.02
Biological process	regulation of transport	24	0.02
Biological process	dorsal/ventral pattern formation	28	0.02
Biological process	detection of visible light	9	0.02
Biological process	regulation of cell morphogenesis	27	0.02
Biological process	cellular response to endogenous stimulus	20	0.02
Biological process	regulation of immune system process	23	0.02
Biological process	regulation of response to external stimulus	10	0.02
Biological process	glycerophospholipid metabolic process	16	0.02
Biological process	cell morphogenesis	78	0.03
Biological process	cellular response to growth factor stimulus	11	0.03
Biological process	cell fate commitment	60	0.03
Biological process	regulation of organ growth	12	0.03
Biological process	pyridine-containing compound metabolic process	8	0.03
Biological process	regulation of G-protein coupled receptor protein signaling pathway	9	0.04
Biological process	cellular biogenic amine metabolic process	9	0.04
Biological process	salivary gland histolysis	17	0.04
Biological process	phototransduction	12	0.04

Biological process	imaginal disc-derived wing morphogenesis	48	0.04
Biological process	enzyme linked receptor protein signaling pathway	28	0.04
Biological process	negative regulation of cell differentiation	22	0.04
Biological process	regulation of BMP signaling pathway	8	0.04
Biological process	fatty acid beta-oxidation	6	0.04
Biological process	protein phosphorylation	38	0.05
Biological process	renal system development	16	0.05
Biological process	morphogenesis of a polarized epithelium	19	0.05

Table S6 Over-represented GO terms among Europe-biased genes.

Ontology	GO Term	Genes	Adj. *P*
Molecular function	electron carrier activity	32	0.0001
Molecular function	oxidoreductase activity, acting on paired donors, with incorporation or reduction of molecular oxygen	31	0.01
Molecular function	cofactor binding	31	0.01
Molecular function	oxidoreductase activity, acting on the CH-OH group of donors, NAD or NADP as acceptor	19	0.01
Molecular function	ATP binding	79	0.02
Molecular function	iron ion binding	28	0.02
Molecular function	transporter activity	99	0.03
Molecular function	disulfide oxidoreductase activity	10	0.03
Cellular component	membrane part	173	0.02
Cellular component	mitochondrial envelope	38	0.02
Cellular component	cytoskeletal part	69	0.03
Cellular component	lipid particle	33	0.04
Biological process	oxidation-reduction process	87	4.6e-08
Biological process	organonitrogen compound metabolic process	84	0.001
Biological process	carboxylic acid metabolic process	49	0.001
Biological process	carbohydrate derivative metabolic process	59	0.01
Biological process	cellular response to stress	56	0.02
Biological process	single-organism transport	138	0.02
Biological process	cellular localization	81	0.03
Biological process	centrosome organization	23	0.03
Biological process	organic substance catabolic process	61	0.04
Biological process	cell redox homeostasis	13	0.04
