# Supplementary material for: Population- and Sex-Biased Gene Expression in the Excretion Organs of Drosophila melanogaster
Source: G3 (Bethesda). 2014 Sep 22;4(12):2307–15. doi: 10.1534/g3.114.013417 (PMC4267927; doi:10.1534/g3.114.013417)
Supplement: Supporting Information [file supp_g3.114.013417_TableS1.pdf]

Table S1   Number of genes meeting various read-count thresholds

Reads	Genes	Sex-biased (female, male)	Population-biased (Africa, Europe)
12	12,547	2,308 (905, 1,403)	2,474 (1,230, 1,244)
20	12,001	2,272 (898, 1,374)	2,459 (1,222, 1,237)
50	10,793	2,098 (858, 1,240)	2,436 (1,206, 1,230)
100	9,785	1,871 (798, 1,073)	2,382 (1,182, 1,200)
150	9,272	1,785 (772, 1,013)	2,340 (1,164, 1,176)

Shown is the total number of genes meeting each read-count threshold, as well as the number of significant sex- and population-biased genes at a FDR of 5%.
