# Supplementary material for: Population- and Sex-Biased Gene Expression in the Excretion Organs of Drosophila melanogaster
Source: G3 (Bethesda). 2014 Sep 22;4(12):2307–15. doi: 10.1534/g3.114.013417 (PMC4267927; doi:10.1534/g3.114.013417)
Supplement: Supporting Information [file supp_g3.114.013417_TableS2.pdf]

Table S2 Number of significant genes detected by different methods

Bias	DESeq2	edgeR (overlap[a])	baySeq (overlap[a])
Sex	2,308	1,249 (85.6%)	557 (98.4%)
Female	905	251 (91.2%)	88 (98.9%)
Male	1,403	998 (84.2%)	469 (98.3%)
Population	2,474	1,015 (87.9%)	496 (97.2%)
Africa	1,230	465 (88.2%)	200 (95.5%)
Europe	1,244	550 (87.6%)	296 (98.3%)

[a] Percent overlap with the significant genes detected by DESeq2.
