# Supplementary material for: Population- and Sex-Biased Gene Expression in the Excretion Organs of Drosophila melanogaster
Source: G3 (Bethesda). 2014 Sep 22;4(12):2307–15. doi: 10.1534/g3.114.013417 (PMC4267927; doi:10.1534/g3.114.013417)
Supplement: Supporting Information [file supp_g3.114.013417_TableS3.pdf]

Table S3 Over-represented GO terms among female-biased genes.

Ontology	GO Term	Genes	Adj. *P*
Molecular function	structural constituent of ribosome	54	1.8e-12
Molecular function	proton-transporting ATPase activity, rotational mechanism	15	1.1e-08
Molecular function	ATP binding	70	9.6e-05
Molecular function	mRNA binding	29	0.001
Molecular function	proton-transporting ATP synthase activity, rotational mechanism	7	0.001
Molecular function	structural constituent of chorion	6	0.001
Molecular function	ATP-dependent RNA helicase activity	11	0.003
Molecular function	hydrogen-exporting ATPase activity, phosphorylative mechanism	8	0.005
Molecular function	actin binding	19	0.01
Molecular function	microtubule binding	14	0.02
Molecular function	aminoacyl-tRNA editing activity	4	0.02
Molecular function	L-amino acid transmembrane transporter activity	5	0.03
Molecular function	symporter activity	14	0.03
Molecular function	protein serine/threonine kinase activity	25	0.03
Molecular function	inorganic anion transmembrane transporter activity	11	0.04
Molecular function	acid phosphatase activity	5	0.04
Cellular component	cytosolic large ribosomal subunit	22	6.0e-10
Cellular component	cytosolic small ribosomal subunit	19	6.0e-10
Cellular component	plasma membrane proton-transporting V-type ATPase complex	10	1.6e-07
Cellular component	microtubule associated complex	50	6.8e-06
Cellular component	lipid particle	34	1.8e-05
Cellular component	vacuolar proton-transporting V-type ATPase, V1 domain	7	0.001
Cellular component	chorion	11	0.001
Cellular component	spindle midzone	6	0.002
Cellular component	nucleolus	21	0.003
Cellular component	mitochondrial matrix	23	0.006
Cellular component	mitochondrial proton-transporting ATP synthase complex, catalytic core F(1)	5	0.01
Cellular component	small-subunit processome	4	0.02
Cellular component	euchromatin	6	0.03
Cellular component	centrosome	14	0.03
Biological process	mitotic spindle elongation	24	5.8e-09
Biological process	ATP hydrolysis coupled proton transport	15	4.7e-07
Biological process	rRNA processing	14	1.3e-05
Biological process	centrosome duplication	20	1.3e-05
Biological process	germ cell development	44	0.0002
Biological process	protein localization	49	0.002
Biological process	asymmetric neuroblast division	11	0.002
Biological process	regulation of cell shape	19	0.004

Biological process	regulation of growth	28	0.005
Biological process	ribosomal small subunit biogenesis	5	0.005
Biological process	protein phosphorylation	33	0.006
Biological process	negative regulation of cellular process	78	0.007
Biological process	actin cytoskeleton organization	27	0.007
Biological process	female germ-line sex determination	4	0.008
Biological process	translational elongation	8	0.008
Biological process	regulation of cellular component size	17	0.01
Biological process	chorion-containing eggshell formation	20	0.01
Biological process	cellular macromolecule localization	39	0.01
Biological process	long-term memory	12	0.01
Biological process	ATP synthesis coupled proton transport	7	0.01
Biological process	ommochrome biosynthetic process	7	0.01
Biological process	regulation of cell differentiation	35	0.01
Biological process	regulation of cellular component biogenesis	22	0.02
Biological process	regulation of cytoskeleton organization	15	0.02
Biological process	L-amino acid transport	4	0.02
Biological process	mitotic cell cycle phase transition	7	0.02
Biological process	establishment of localization in cell	53	0.02
Biological process	pupariation	5	0.03
Biological process	regulation of DNA replication	6	0.03
Biological process	cellular component assembly involved in morphogenesis	23	0.03
Biological process	amino acid transmembrane transport	8	0.03
Biological process	positive regulation of cellular component organization	16	0.03
