# Supplementary material for: Population- and Sex-Biased Gene Expression in the Excretion Organs of Drosophila melanogaster
Source: G3 (Bethesda). 2014 Sep 22;4(12):2307–15. doi: 10.1534/g3.114.013417 (PMC4267927; doi:10.1534/g3.114.013417)
Supplement: Supporting Information [file supp_g3.114.013417_TableS4.pdf]

Table S4 Over-represented GO terms among male-biased genes.

Ontology	GO Term	Genes	Adj. *P*
Molecular function	electron carrier activity	42	5.2e-10
Molecular function	manganese ion binding	12	4.1e-06
Molecular function	iron ion binding	37	5.4e-06
Molecular function	glutathione transferase activity	17	7.3e-06
Molecular function	acyl-CoA dehydrogenase activity	10	2.2e-05
Molecular function	oxidoreductase activity, acting on paired donors, with incorporation or reduction of molecular oxygen	37	4.6e-05
Molecular function	flavin adenine dinucleotide binding	19	5.4e-05
Molecular function	oxidoreductase activity, acting on the CH-OH group of donors, NAD or NADP as acceptor	23	7.5e-05
Molecular function	NAD binding	13	0.003
Molecular function	aminopeptidase activity	13	0.01
Molecular function	carbohydrate kinase activity	8	0.01
Molecular function	hydro-lyase activity	13	0.02
Molecular function	oxidoreductase activity, acting on the aldehyde or oxo group of donors, NAD or NADP as acceptor	11	0.02
Molecular function	long-chain fatty acid transporter activity	5	0.03
Molecular function	disulfide oxidoreductase activity	10	0.03
Cellular component	lipid particle	44	5.9e-06
Cellular component	mitochondrial matrix	35	2.6e-05
Cellular component	endoplasmic reticulum part	23	0.02
Cellular component	coated vesicle membrane	9	0.03
Cellular component	peroxisome	12	0.03
Cellular component	Golgi membrane	11	0.04
Biological process	fatty acid beta-oxidation	10	4.6e-06
Biological process	glutathione metabolic process	18	5.8e-06
Biological process	protein folding	27	3.8e-05
Biological process	glycolysis	12	0.0002
Biological process	dicarboxylic acid metabolic process	10	0.02
Biological process	branched-chain amino acid metabolic process	5	0.03
Biological process	glycerol ether metabolic process	7	0.03
