# Supplementary material for: Population- and Sex-Biased Gene Expression in the Excretion Organs of Drosophila melanogaster
Source: G3 (Bethesda). 2014 Sep 22;4(12):2307–15. doi: 10.1534/g3.114.013417 (PMC4267927; doi:10.1534/g3.114.013417)
Supplement: Supporting Information [file supp_g3.114.013417_TableS5.pdf]

Table S5 Over-represented GO terms among Africa-biased genes.

Ontology	GO Term	Genes	Adj. *P*
Molecular function	proton-transporting ATPase activity, rotational mechanism	15	1.7e-06
Molecular function	heme binding	30	7.3e-05
Molecular function	iron ion binding	30	0.002
Molecular function	anion binding	117	0.002
Molecular function	cofactor binding	30	0.01
Molecular function	nucleotide binding	122	0.02
Molecular function	protein kinase activity	40	0.04
Molecular function	oxidoreductase activity, acting on the CH-OH group of donors, NAD or NADP as acceptor	17	0.04
Molecular function	glutathione transferase activity	11	0.04
Cellular component	vacuolar proton-transporting V-type ATPase, V1 domain	9	8.9e-05
Biological process	ATP hydrolysis coupled proton transport	16	7.5e-06
Biological process	carbohydrate metabolic process	48	0.0001
Biological process	male courtship behavior, veined wing generated song production	10	0.001
Biological process	chemical homeostasis	25	0.001
Biological process	negative regulation of signal transduction	35	0.001
Biological process	neuron development	83	0.001
Biological process	regulation of organ morphogenesis	19	0.004
Biological process	glutathione metabolic process	13	0.007
Biological process	regulation of neurogenesis	24	0.01
Biological process	visual perception	11	0.02
Biological process	taxis	43	0.02
Biological process	regulation of transport	24	0.02
Biological process	dorsal/ventral pattern formation	28	0.02
Biological process	detection of visible light	9	0.02
Biological process	regulation of cell morphogenesis	27	0.02
Biological process	cellular response to endogenous stimulus	20	0.02
Biological process	regulation of immune system process	23	0.02
Biological process	regulation of response to external stimulus	10	0.02
Biological process	glycerophospholipid metabolic process	16	0.02
Biological process	cell morphogenesis	78	0.03
Biological process	cellular response to growth factor stimulus	11	0.03
Biological process	cell fate commitment	60	0.03
Biological process	regulation of organ growth	12	0.03
Biological process	pyridine-containing compound metabolic process	8	0.03
Biological process	regulation of G-protein coupled receptor protein signaling pathway	9	0.04
Biological process	cellular biogenic amine metabolic process	9	0.04
Biological process	salivary gland histolysis	17	0.04
Biological process	phototransduction	12	0.04

Biological process	imaginal disc-derived wing morphogenesis	48	0.04
Biological process	enzyme linked receptor protein signaling pathway	28	0.04
Biological process	negative regulation of cell differentiation	22	0.04
Biological process	regulation of BMP signaling pathway	8	0.04
Biological process	fatty acid beta-oxidation	6	0.04
Biological process	protein phosphorylation	38	0.05
Biological process	renal system development	16	0.05
Biological process	morphogenesis of a polarized epithelium	19	0.05
