# Supplementary material for: Population- and Sex-Biased Gene Expression in the Excretion Organs of Drosophila melanogaster
Source: G3 (Bethesda). 2014 Sep 22;4(12):2307–15. doi: 10.1534/g3.114.013417 (PMC4267927; doi:10.1534/g3.114.013417)
Supplement: Supporting Information [file supp_g3.114.013417_TableS6.pdf]

Table S6 Over-represented GO terms among Europe-biased genes.

Ontology	GO Term	Genes	Adj. *P*
Molecular function	electron carrier activity	32	0.0001
Molecular function	oxidoreductase activity, acting on paired donors, with incorporation or reduction of molecular oxygen	31	0.01
Molecular function	cofactor binding	31	0.01
Molecular function	oxidoreductase activity, acting on the CH-OH group of donors, NAD or NADP as acceptor	19	0.01
Molecular function	ATP binding	79	0.02
Molecular function	iron ion binding	28	0.02
Molecular function	transporter activity	99	0.03
Molecular function	disulfide oxidoreductase activity	10	0.03
Cellular component	membrane part	173	0.02
Cellular component	mitochondrial envelope	38	0.02
Cellular component	cytoskeletal part	69	0.03
Cellular component	lipid particle	33	0.04
Biological process	oxidation-reduction process	87	4.6e-08
Biological process	organonitrogen compound metabolic process	84	0.001
Biological process	carboxylic acid metabolic process	49	0.001
Biological process	carbohydrate derivative metabolic process	59	0.01
Biological process	cellular response to stress	56	0.02
Biological process	single-organism transport	138	0.02
Biological process	cellular localization	81	0.03
Biological process	centrosome organization	23	0.03
Biological process	organic substance catabolic process	61	0.04
Biological process	cell redox homeostasis	13	0.04
